# Neuroimmune Correlates of HIV and Marijuana Use: Peripheral Biomarkers and Cognitive Function

**DOI:** 10.1007/s11481-026-10299-6

**Published:** 2026-06-23

**Authors:** Michaela E. Larson, E. Alfonso Romero-Sandoval, Sheri L. Towe, Adam W. Carrico, Christina S. Meade

**Affiliations:** 1 Robert Stempel College of Public Health and Social Work, Florida International University, Miami, FL USA; 2 Department of Anesthesiology, Wake Forest University School of Medicine, Winston-Salem, NC USA; 3 Department of Translational Neuroscience, Wake Forest University School of Medicine, Winston-Salem, NC USA

**Keywords:** HIV-associated immune activation, Marijuana use, Inflammatory biomarkers, Cannabinoids, Neuroimmune function

## Abstract

People with HIV (PWH) exhibit persistent immune activation despite suppressive antiretroviral therapy, contributing to neurocognitive vulnerability. Marijuana use is common among PWH and may influence inflammatory pathways, but its in vivo immunologic effects in treated HIV remain unclear. In this cross-sectional study (Durham, NC; 2021–2023), 238 adults with and without HIV were grouped by chronic marijuana use. Soluble immune biomarkers were measured in plasma/serum, and log-transformed outcomes were analyzed using hierarchical multivariable regression. Seven biomarkers showed significant model fit: sCD163, IFN-γ, TNF-α, TNF-RII, CXCL10, CCL4, and VCAM-1. Among HIV-negative participants, marijuana use was linked to reduced CXCL10, suggesting disruption of IFN-γ-dependent chemotactic signaling. In contrast, HIV infection in the absence of marijuana use was associated with a broad, multi-pathway inflammatory profile, including TNF signaling, interferon activation, monocyte/macrophage activation, and endothelial recruitment. Among PWH, marijuana use did not modulate these effects, although CCL4 and VCAM-1 were not significantly elevated in this group compared to controls. However, these markers did not differ between marijuana-using and non-using PWH, suggesting a potential divergence from the broader inflammatory profile rather than a clear marijuana-associated attenuation. Cognitive analyses demonstrated a modest inverse association between memory performance and TNF-related markers, indicating that HIV-associated inflammatory signaling may relate to memory function, whereas marijuana-related cognitive effects require further study.

**Graphical Abstract:**

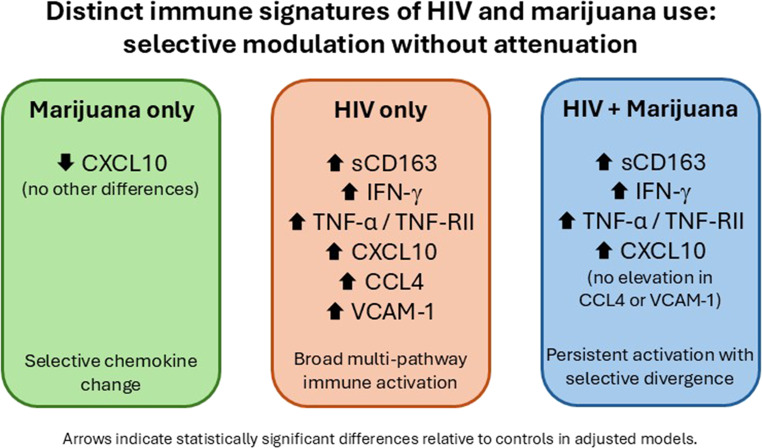

**Supplementary Information:**

The online version contains supplementary material available at 10.1007/s11481-026-10299-6.

## Introduction

Immune activation and inflammation are hallmarks of HIV pathophysiology and contribute to central nervous system (CNS) complications such as cognitive impairment (Chan and Spudich 2024; O’Connor et al. 2023; Spudich 2016). Acute HIV infection triggers a range of immune responses, including elevations in circulating cytokines and activation of natural killer cells (Kazer et al. 2020). HIV-induced inflammation is paradoxical in that immune activation predicts disease progression (Claiborne et al. 2015). Combination antiretroviral therapy (cART) dramatically reduces immune activation and systemic inflammation in people with HIV (PWH), but not to pre-infection levels (Hileman and Funderburg 2017).

This persistent inflammation has important implications for neuroimmune outcomes. Neurocognitive difficulties remain common among virally suppressed PWH, and often include vulnerabilities in learning and memory, alongside other cognitive systems (Chan and Valcour 2021; Elendu et al. 2023). Neurocognitive risk in treated HIV is multifactorial and has been linked to ongoing immune activation/inflammation, and common comorbid factors (e.g., depression, cardiometabolic risk, substance use) (McIntosh et al. 2021; Tedaldi et al. 2015). HIV-infected monocytes and T-cells traffic from the periphery into the brain, a key reservoir for latent HIV, creating neurotoxic environments of resident brain cells (Hong and Banks 2015; León-Rivera et al. 2021; Wahl and Al-Harthi 2023; Williams et al. 2014). Disruption of the blood-brain-barrier enables greater infiltration of immune cells and inflammatory mediators. Activated microglia amplify this response by releasing proinflammatory cytokines such as interleukin (IL) -1, tumor necrosis factor alpha (TNF-α), interferon gamma (IFN-γ),-and monocyte chemoattractant protein (MCP)-1 (Williams and Naudé 2024). These cytokines further initiate inflammatory cascades that are thought to promote neuronal degeneration in key brain regions associated with emotional processing, executive functioning, and memory (Rubin et al. 2018; Shan et al. 2024). Although numerous studies have sought to identify predictive biomarkers of neuropsychiatric comorbidities in PWH, it is increasingly recognized that a distinct combination of biomarkers are needed for more accurate predictions for neuropsychiatric conditions (Anderson et al. 2021; Petersen et al. 2023; Rubin et al. 2025).

Marijuana use is disproportionately prevalent among PWH. Across samples, an estimated 20–60% of PWH report current marijuana use, and nearly half of these individuals report daily use (D’Souza et al. 2012; Okafor et al. 2017; Parisi et al. 2023). Comparatively, only 10–15% of the general United States adult population reports current marijuana use (Centers for Disease Control and Prevention, 2025; Mattingly et al. 2024). Elevated marijuana use among PWH may be driven by the need for HIV- and cART-related symptom relief, stress reduction, and appetite stimulation (Bruce et al. 2020; D’Souza et al. 2012; Haug et al. 2017; Ware et al. 2010). Although the therapeutic benefits are often endorsed as primary reasons for marijuana use among PWH, the prevalence of medically prescribed marijuana use remains low, around 7% (Costiniuk et al. 2019; D’Souza et al. 2012; Montgomery et al. 2019). Despite self-reported symptom relief, marijuana use in PWH has been associated with both clinically beneficial and detrimental health outcomes. While some studies suggest that marijuana use may reduce systemic inflammation and neuropathic pain, others highlight associations of marijuana use with increased depressive symptoms, neurocognitive impairment, and cardiovascular disease risk (Costiniuk et al. 2019; Lorenz et al. 2017; Montgomery et al. 2019; Thames et al. 2016). Given the high prevalence of marijuana use among PWH and its variable clinical effects, further research is needed to clarify how the biological mechanisms of marijuana’s active components relate to neurocognitive functioning.

The biologically active components of marijuana, primarily Δ9-tetrahydrocannabinol (THC) and cannabidiol (CBD), exert their effects through the human endocannabinoid system by binding to cannabinoid receptors on neuronal and glial cells in the CNS and on immune cells and lymphoid tissues in the periphery (National Academies of Sciences, 2017). The psychotropic effects of THC result from binding at cannabinoid receptor type 1 (CB1), which are highly concentrated in sub-cortical and prefrontal regions in brain networks responsible for learning, memory, and cognitive processing (Bloomfield et al. 2019; Burggren et al. 2019; Mackie 2008). Chronic THC exposure induces CB1 downregulation, and reductions in grey matter volume have been observed in CB1-enriched regions, possibly reflecting neuronal loss, decreased cell size, and lower CB1 density (Filbey et al. 2014). CBD is non-intoxicating and exerts primarily immunomodulatory (often anti-inflammatory) effects through its low-affinity, allosteric modulation of CB1, CB2, and non-cannabinoid receptors (Martinez Naya et al. 2023; Nichols and Kaplan 2020). Beyond the CNS effects, both THC and CBD interact with CB1 and CB2 receptors on peripheral immune cells. Activation of CB2 is key to the immunosuppressive effects of phytocannabinoids, while CB1 expression can promote inflammation (Turcotte et al. 2016). CBD further suppresses immune activation by antagonizing CB1 and CB2 receptor activation with ligands to inhibit immune cell migration and reduce endocannabinoid breakdown (Ligresti et al. 2016). In vitro studies have demonstrated that exogenous cannabinoids can downregulate pro-inflammatory cytokines such as TNF-α, IFN-γ, and IL-1 (Henshaw et al. 2021). However, the translational and clinical relevance of these effects in vivo remains unclear.

While clinical studies examining the effects of marijuana use on immune function remain limited and nuanced, population-based studies have reported no or marginally significant differences in pro-inflammatory markers such as C-reactive protein (CRP) and IL-6, while others have associated marijuana use with higher levels of anti-inflammatory cytokines (e.g., IL-20), lower levels of pro-inflammatory cytokines (e.g., IL-16), and decreased microbial translocation (Alshaarawy et al. 2019; Ellis et al. 2020; Krsak et al. 2021; Okafor et al. 2024). Furthermore, some studies suggest that PWH on suppressive cART who use marijuana may have reduced frequencies of activated T-cells and inflammatory monocytes, suggesting decreased immune activation (Manuzak et al. 2018). Further research is needed to examine associations of chronic marijuana use with markers of immune activation and inflammation that amplify risk for HIV-associated comorbidities such as cardiovascular disease.

This cross-sectional study investigated the distinct and combined associations of HIV and chronic marijuana use with soluble biomarkers of immune activation, inflammation, chemokine signaling, and vascular injury. We hypothesized that marijuana use would diminish HIV-associated elevations in immune activation and systemic inflammation. Among marijuana users, we examined the dose-response associations of cannabinoid metabolite levels with these biomarkers. We hypothesized that lower CBD and higher THC levels would be associated with that greater activation and inflammatory signaling. Finally, we examined the extent to which biomarker profiles were associated with cognitive functioning, specifically in the domains of learning and memory domains.

## Methods

This study is a baseline analysis of biospecimen and self-reported survey data collected as part of a longitudinal cohort study that focused on the distinct and combined associations of treated HIV and marijuana use on inflammation and cognition. Data was collected from September 2021 to June 2023 in Durham, North Carolina.

### Participants

This study enrolled adults aged 25–59 years with and without HIV who either used marijuana (MJ+) or did not use marijuana (MJ-), resulting in four intact groups (HIV-MJ-, HIV-MJ+, HIV + MJ-, HIV + MJ+). PWH met the following criteria: (1) non-perinatal infection; (2) receipt of cART as first-line treatment; (3) current cART use; and (4) plasma HIV RNA levels of < 200 copies/mL at time of enrollment. Participants who used marijuana met the following criteria: (1) ≥4 days of marijuana use in the past 30 days; (2) ≥1 year of regular lifetime marijuana use, defined as ≥3 times a week for ≥6 months; and (3) a THC-positive urine drug screen. Participants who did not use marijuana met the following criteria: (1) 0 days of marijuana use in the past 30 days; (2) a THC-negative urine drug screen; and (3) no history of regular marijuana use within the past 10 years.

Alcohol and nicotine use were permitted for all groups. For illicit drugs other than marijuana, participants were excluded if they had a positive urine drug screen, reported ≥2 days of use in the past 30 days, reported regular use in the past 2 years, and/or reported ≥5 years of regular lifetime use. Additional exclusion criteria were: limited English proficiency, inability to read, history of severe head trauma (e.g., traumatic brain injury), unstable or serious neurological disorders requiring treatment or outpatient evaluation within the past 5 years (e.g., seizure disorders, dementia or Alzheimer’s disease), acutely symptomatic CNS infection or history of such infection with residual symptoms (e.g., memory loss, seizures), severe mental illness requiring inpatient hospitalization in the past year, current antipsychotic or mood stabilizing medication use, acute psychiatric symptoms, relevant systemic autoimmune disease (e.g., lupus, rheumatoid arthritis), current leukemia or lymphoma diagnosis, history of organ or bone marrow transplant, current dialysis, and any history of receipt of immunomodulatory therapy, including chemotherapy, blood transfusion, and radiation treatment.

### Procedures

Participants were recruited via advertisements and flyers in local community-based organizations, websites, and medical clinics. Following a brief telephone interview to assess preliminary eligibility, participants provided written informed consent and completed an in-person eligibility screening that included questionnaires and clinical interviews on substance, medical and psychiatric history, urine drug testing, urine pregnancy testing (for participants of child-bearing potential), and HIV screening. Medical records were reviewed to ensure no exclusionary medical conditions were present. Eligible participants then completed another visit that included peripheral blood sample collection, neuropsychological testing, and additional interviews and questionnaires. All procedures were approved by the Duke Health Institutional Review Board.

### Measures

#### HIV Status

For participants with known HIV diagnosis, HIV-positive status was verified via medical record review. HIV viral load was confirmed at the baseline visit as < 200 copies/mL via quantitative PCR testing through Quest Diagnostics (test code 0040085), along with a lymphocyte panel (test code 0007197). For all others, a rapid HIV-1/2 antibody test (OraQuick ADVANCE^®^ or Chembio SURE CHECK^®^) was conducted using a droplet of whole blood collected by pinprick. All participants who were screened had a non-reactive test result.

#### Marijuana and Other Substance Use

At the baseline visit, participants self-reported days of use in the past 30 days for all substances, including marijuana, alcohol, and nicotine. For marijuana, they also reported the routes of administration and estimates of the average duration of intoxication (in hours) on days they used. Duration, frequency, and quantity of lifetime marijuana use were assessed using the Lifetime Drug Use Questionnaire (Czermak et al. 2005). Marijuana use disorder was assessed via the Structured Clinical Interview for DSM (SCID) (First et al. 2015). Metabolites of marijuana use, specifically 7-carboxy-CBD (7-COOH-CBD) and 11-nor-9-carboxy-Δ9-THC (11-COOH-THC), from venous blood samples were quantified using a validated liquid chromatography–tandem mass spectrometry (LC-MS/MS) assay (Proença et al. 2023). Marijuana use metabolites were also dichotomized to reflect any versus no detection of the metabolite. One participant in the HIV + MJ- group tested positive for THC and was removed from the analysis.

#### Clinical Variables

##### Depression

Depression severity was assessed via the depression sub-scale of the Symptom Checklist-90 Revised (SCL-90-R) (Derogatis and Savitz 1999). Scores represent the mean of 13 items.

##### Kidney and Liver Function

Participants provided blood samples that were sent to Quest Diagnostics for Complete Blood Count (CBC, test code 6399) and Comprehensive Metabolic Panel (CMP, test code 10231). Kidney function was assessed using estimated glomerular filtration rate (eGFR) as part of the CMP. Following guidelines from the National Kidney Foundation, kidney function was dichotomized into normal kidney function (eGFR ≥ 90) and abnormal function (eGFR < 90) (Lu et al. 2023). Liver function was assessed via the fibrosis-4 (FIB-4) index. FIB-4 scores are calculated from participants’ age, aspartate transaminase (AST), alanine transaminase (ALT), and platelet count from the CMP and CBC with the following equation: FIB-4 = [(Age × AST)/(Platelets × √ALT)]. Outcomes were dichotomized into risk categories to reflect low risk (FIB-4 < 1.3) and elevated risk (FIB-4 ≥ 1.3) (Schreiner et al. 2022).

##### Cardiometabolic Function

Cardiometabolic function was assessed via medical record abstraction as ever having been diagnosed with high cholesterol (yes/no).

#### Cognitive Function

The following seven domains of cognitive function were assessed via standardized neuropsychological tests: executive function, attention, learning, memory, information processing speed, motor function, and fluency. A summary of assessments used for each domain can be found in Supplemental Table 1. Raw scores for each test were converted to demographically adjusted T scores using the most updated scoring software available from the test publisher. T scores are standardized (M = 50, SD = 10), with scores of 40 and 30 corresponding to 1 and 2 standard deviations below the normative mean, respectively. We computed the domain T score by taking an average of all the tests within each domain.

#### Other Participant Characteristics

Demographic information including sex at birth, age, gender, race/ethnicity, education, and employment was collected via self-reported surveys.

#### Peripheral Biomarkers

Non-fasting blood samples were collected by peripheral venipuncture. Plasma and serum were purified using standard procedures, and then supernatant was pipetted into aliquots and stored at -80 °C until time of assay. Biomarkers were assayed on the MesoScale Discovery Multi-array platform using commercially available enzyme-linked immunosorbent assay (ELISA) kits. All analytes were measured in duplicate following manufacturers’ instructions. Control Human Plasma was included on all plates to assess inter-assay variability. Multiplex kits from MesoScale Discovery (Rockville, MD) were used to measure concentrations of: IFN-γ, IL-1β, IL-2, IL-4, IL-6, IL8, IL-10, IL-12p70, IL-13, and TNF-α (VPLEX Proinflammatory Panel, Cat#K15049D); Eotaxin, CXCL10 (IP-10), MCP-1, CCL4 (MIP1β), MDC (custom VPLEX Chemokine Panel, Cat#K15047D); MMP-9, TNFRI, TNFRII (custom UPLEX 3-plex, Cat#K151AEM-1); LBP (RPLEX LBP, Cat#K151K5R); and CRP, ICAM-1, and VCAM-1 (custom VPLEX Vascular Injury, Cat#K15198D). Single ELISA assays from R&D (Minneapolis, MN) were used for the following analytes: sCD14 (Cat#DC140) and sCD163 (Cat#DC1630). Laboratory personnel were blinded to all participant characteristics, including demographic information and study group.

### Data Analysis

#### Descriptive Statistics

Comparison of the HIV/MJ groups across demographic, HIV-related, substance use, kidney, liver, cardiometabolic, and cognition factors were assessed via Chi-square and Fisher’s Exact tests for categorical variables, and analysis of variance (ANOVA) or Kruskal-Wallis tests for continuous variables, as appropriate. When global tests were significant, post-hoc pairwise comparisons were done to examine differences between the groups, including Dunn’s test for non-parametric continuous outcomes, t-tests for normally distributed continuous outcomes, and pairwise Fisher’s Exact tests for categorical outcomes. All post-hoc tests were corrected for multiple comparison with Bonferroni correction.

#### Differences in Peripheral Biomarkers Across Groups

Peripheral biomarkers were standardized and normalized prior to analysis. Biomarkers where most cases (> 50%) were below the lower limit of detection (LLOD; the calculated concentration corresponding to a signal 2.5 standard deviations above background) were excluded. Five biomarkers met this exclusion criterion: IL-1β, IL-2, IL-4, IL-12p70, and IL-13, leaving 21 for analysis. For all remaining analytes, less than 0.5% of samples were below the LLOD. To improve the normality of the peripheral biomarkers, each was log transformed (log 10). Extreme outliers, defined as >3 standard deviations from the mean, were excluded.

The analysis of differences in peripheral biomarkers between groups was assessed via univariable and hierarchical multivariable linear regression. Each peripheral biomarker was modeled separately. The hierarchical multivariable linear regressions proceeded in two steps. First, covariates known to affect inflammatory and metabolic peripheral biomarkers were entered. These were age, sex, alcohol use, nicotine use, kidney function, liver function, cardiometabolic function, and depression. The HIV/MJ grouping variable was then entered as a second step in the models, with the HIV-MJ- group set as the reference. Raw *p*-values were false discovery rate (FDR) corrected with the Benjamini-Hochberg (BH) procedure. For model comparison (step 1 versus step 2) we compared with change in R^2^ between models for each biomarker.

To visualize group differences in biomarkers where the HIV/MJ grouping remained a significant predictor in the hierarchical models, we generated boxplots stratified by group. Pairwise comparisons between groups were conducted using estimated marginal means from the multivariable models (step 2 – fully adjusted). The *p*-values from the pairwise contrasts were FDR corrected for multiple comparisons using the BH procedures. Adjusted significance levels were annotated on the plots.

#### Exploratory Correlations

Lastly, exploratory analyses using Pearson correlation were done to examine the relationship between the significant peripheral biomarkers, and constructs of cognitive function. Among participants who used marijuana (i.e., HIV-/MJ + and HIV+/MJ-), we examined the correlation between the significant peripheral biomarkers and cannabinoid metabolites. Two-tailed p-values were derived from a t-distribution with n-2 degrees of freedom. All data analysis was performed in RStudio version 4.5.0.

## Results

### Participants Characteristics

Characteristics of the 238 participants by HIV and marijuana (MJ) status are shown in Table 1. Groups did not differ in sex, age, or race distribution. Overall, 81% (*n* = 177) identified as male, the mean age was 41 years (SD = 8), and 68% (*n* = 149) identified as Black or African American. Educational attainment differed across groups, with the HIV + MJ+ group reporting fewer years of education than the HIV-MJ- and HIV + MJ- groups.

**Table 1:** Cohort characteristics by HIV/MJ groups (*N* = 238)

Demographics	HIV-MJ- (*N* = 60)	HIV-MJ+ (*N* = 60)	HIV + MJ- (*N* = 60)	HIV + MJ+ (*N* = 58)	*p*-value
Sex					0.937
Male	44 (73.3%)	44 (73.3%)	44 (73.3%)	45 (77.6%)	
Female	16 (26.7%)	16 (26.7%)	16 (26.7%)	13 (22.4%)	
Age					0.132
Mean (SD)	41.3 (8.00)	38.5 (7.22)	41.3 (7.92)	41.4 (8.53)	
Race					0.696
Black or African American	40 (66.7%)	40 (66.7%)	33 (55.0%)	36 (62.1%)	
White	17 (28.3%)	14 (23.3%)	19 (31.7%)	16 (27.6%)	
Other or Mixed	3 (5.0%)	6 (10.0%)	8 (13.3%)	6 (10.3%)	
Years of Education					**< 0.001**
Mean (SD)	15.7 (2.01)^a^	14.7 (2.09)	15.9 (2.46)^b^	14.0 (2.11)^a, b^	
HIV-Related Factors					
Years Living with HIV					0.215
Mean (SD)	-	-	12.0 (7.01)	13.8 (8.11)	
Years on ART					0.566
Mean (SD)	-	-	10.5 (6.36)	11.6 (8.00)	
Absolute CD4 count					0.581
Mean (SD)	-	-	818.45 (296.58)	856.79 (346.33)	
Absolute CD8 count					0.581
Mean (SD)	-	-	904.92 (414.21)	943.34 (407.96)	
CD4:CD8 Ratio					0.938
Mean (SD)	-	-	1.07 (0.597)	1.07 (0.637)	
Marijuana Use-Related Factors					
Days of Use in the Past 30 Days					**0.044**
Mean(SD)	-	24.4 (7.58)	-	25.9 (7.81)	
Route of Administration^1^					0.655
Inhalation Only	-	39 (65.0%)	-	41 (71.9%)	
Oral Only	-	2 (3.3%)	-	1 (1.8%)	
Both Inhalation and Oral		19 (31.7%)		15 (26.3%)	
Hours per Day ‘High’					0.422
Mean(SD)	-	5.08 (3.52)	-	5.02 (4.17)	
Marijuana Use Disorder, Past 12 Months					0.193
No	-	22 (36.7%)	-	29 (50.0%)	
Yes	-	38 (63.3%)	-	29 (50.0%)	
7-carboxy-CBD					0.717
Not Detected	-	55 (91.7%)	-	55 (94.8%)	
Detected	-	5 (8.3%)	-	3 (5.3%)	
11-nor-9-carboxy-Δ9-THC					0.715
Not Detected	-	3 (5.0%)	-	4 (6.9%)	
Detected	-	57 (95.0%)	-	54 (93.1%)	
Substance Use					
Days of Alcohol Use in the Past 30 Days					0.073
Mean (SD)	3.57 (5.53)	6.43 (8.20)	4.08 (6.11)	6.26 (7.98)	
Any Nicotine Use in the Past 30 Days					**< 0.001**
No	55 (91.7%)	35 (58.3%)	56 (93.3%)	27 (46.6%)	
Yes	5 (8.3%)^a, b^	25 (41.7%)^a, c^	4 (6.7%)^c, d^	31 (53.4%)^b, d^	
Comorbidities					
Depression (symptom checklist score)					**0.025**
Mean (SD)	0.32 (0.49)^a^	0.52 (0.53)^a^	0.31 (0.42)	0.51 (0.57)	
Kidney Function					0.086
Normal (eGFR ≥ 90)	29 (50.0%)	31 (52.5%)	19 (31.7%)	22 (38.6%)	
Abnormal (eGFR < 90)	29 (50.0%)	28 (47.5%)	40 (66.7%)	35 (61.4%)	
Liver Function					0.194
Low Risk (FIB-4 < 1.3)	57 (95.0%)	57 (95.0%)	58 (96.7%)	50 (86.2%)	
Elevated Risk (FIB-4 ≥ 1.3)	3 (5.0%)	2 (3.3%)	2 (3.3%)	7 (12.1%)	
High Cholesterol					0.342
No	42 (71.2%)	45 (83.3%)	46 (76.7%)	48 (82.8%)	
Yes	17 (28.8%)	9 (16.7%)	14 (23.3%)	10 (17.2%)	
Cognitive Function					
Executive Function					0.136
Mean (SD)	52.9 (5.88)	50.5 (6.65)	51.3 (6.28)	50.8 (5.58)	
Attention					0.121
Mean (SD)	53.0 (7.23)	52.4 (7.20)	50.3 (8.51)	50.4 (7.48)	
Learning					**0.001**
Mean (SD)	49.2 (8.63)^a^	46.1 (9.63)	45.1 (10.4)	42.3 (8.44)^a^	
Memory					**0.006**
Mean (SD)	51.0 (8.44)^a^	48.5 (9.89)	47.7 (10.5)	44.7 (9.71)^a^	
Information Processing Speed					0.782
Mean (SD)	49.4 (8.73)	48.0 (8.15)	49.0 (9.05)	48.2 (8.28)	
Motor Function					0.528
Mean (SD)	46.0 (7.70)	45.4 (8.06)	45.6 (8.18)	43.9 (8.77)	
Fluency					0.154
Mean (SD)	52.6 (6.73)	53.2 (7.47)	51.1 (8.43)	50.5 (6.13)	

Note: Groups that share a superscript (e.g., a, b, c, d) differ significantly in Bonferroni corrected post-hoc test

^1^Missing for 1 participant

Among MJ+ participants, the mean number of days of use in the past 30 days was slightly higher in the HIV + MJ+ group (*m* = 26 days, *sd* = 8 days) than the HIV-MJ+ group (*m* = 24 days, *sd* = 8 days, *p* = 0.044). Daily use was nearly universal (98% vs. 100%, respectively). The most common route of marijuana administration was inhalation: 65% reported only inhalation, 32% reported both inhalation and oral, and 3% reported only oral. They reported an average of 5 h per day “high” from marijuana (*sd* = 4). Over half (57%) met criteria for cannabis use disorder in the past 12 months; there were no differences by HIV status. Consistent with self-reports, most MJ+ participants (94%) had detectable 11-COOH-THC, whereas 7-COOH-CBD was detected in only 7% of participants.

Nicotine use in the past 30 days differed significantly across groups, with MJ+ groups reporting higher rates than MJ– groups. Depression scores also differed, with higher mean scores among MJ+ groups. For cognitive function, there were significant group differences for learning and memory, with the mean T score being highest among the HIV-MJ- group and lowest among the HIV + MJ+ group. Although all groups performed within the normative range on average, differences of > 5 points on T scores (~ 0.5 SD) reflect moderate group-level effects and may be clinically relevant insofar as they shift the distribution of performance toward lower functioning. No group differences were observed in alcohol use, kidney function, liver function, or cardiometabolic indicators. Among PWH, clinical HIV characteristics including years since diagnosis, years on cART, CD4 and CD8 T‑cell counts, and CD4:CD8 ratio did not differ by MJ status.

### Peripheral Biomarker Differences Across Groups

Twenty-one peripheral biomarkers were assayed. Univariable linear regression models including HIV/MJ group as the sole predictor identified nine biomarkers (43%) significant at FDR-corrected *p* < 0.05 (Table 2). In hierarchical multivariable models adjusting for age, sex, alcohol use, nicotine use, kidney and liver function, cardiometabolic function, and depression, seven biomarkers (33%) remained significant at FDR-corrected *p* < 0.05 for the added contribution of HIV/MJ grouping: sCD163, IFN-γ, TNF-α, TNF-RII, CXCL10, CCL4, and VCAM-1. Full model coefficients for these seven biomarkers are presented in Supplementary Table 2.

**Table 2:** Results from univariable and hierarchical multivariable linear regressions

Biomarker	Univariable Linear Regression		Hierarchical Multivariable Linear Regression^b^		
	Omnibus^a^ *p*-value	FDR-corrected Omnibus^a^ *p*-value	ΔR^2^	Model Δ *p*-value (Step 2 vs. Step 1^c^)	Model Δ FDR-corrected *p*-value (Step 2 vs. Step 1^c^)
sCD163	< 0.001	**< 0.001**	0.08	< 0.001	**0.002**
IFN-gamma	0.001	**0.004**	0.06	0.003	**0.013**
IL-10	0.128	0.207	0.01	0.534	0.700
IL-6	0.430	0.444	0.03	0.065	0.153
IL-8	0.198	0.297	0.02	0.196	0.373
TNF-alpha	< 0.001	**< 0.001**	0.09	< 0.001	**0.001**
TNF- RI	0.250	0.350	0.01	0.752	0.878
TNF- RII	< 0.001	**< 0.001**	0.07	0.001	**0.003**
Eotaxin	0.312	0.364	0.00	0.969	0.969
CXCL10	< 0.001	**< 0.001**	0.22	< 0.001	**< 0.001**
MCP-1	0.012	**0.028**	0.03	0.074	0.155
MDC	0.278	0.364	0.00	0.896	0.969
CCL4	0.003	**0.008**	0.05	0.005	**0.017**
MMP-9	0.308	0.364	0.01	0.389	0.544
LBP	0.444	0.444	0.01	0.341	0.511
GFAP	0.104	0.182	0.00	0.964	0.969
NfL	0.034	0.071	0.01	0.604	0.746
CRP	0.444	0.444	0.02	0.264	0.462
ICAM-1	0.002	**0.007**	0.04	0.449	0.118
VCAM-1	0.001	**0.005**	0.06	0.006	**0.018**
sCD14	0.068	0.131	0.02	0.319	0.511

^a^HIV/MJ group Type III comparison

^b^Predictors: HIV/MJ group, age, sex at birth, nicotine use, alcohol use, kidney function, liver function, cardiometabolic function, depression

^c^Step 1: covariates only; Step 2: covariates with HIV/MJ group; *p*-value from F-test with FDR correction

**Fig. 1:**
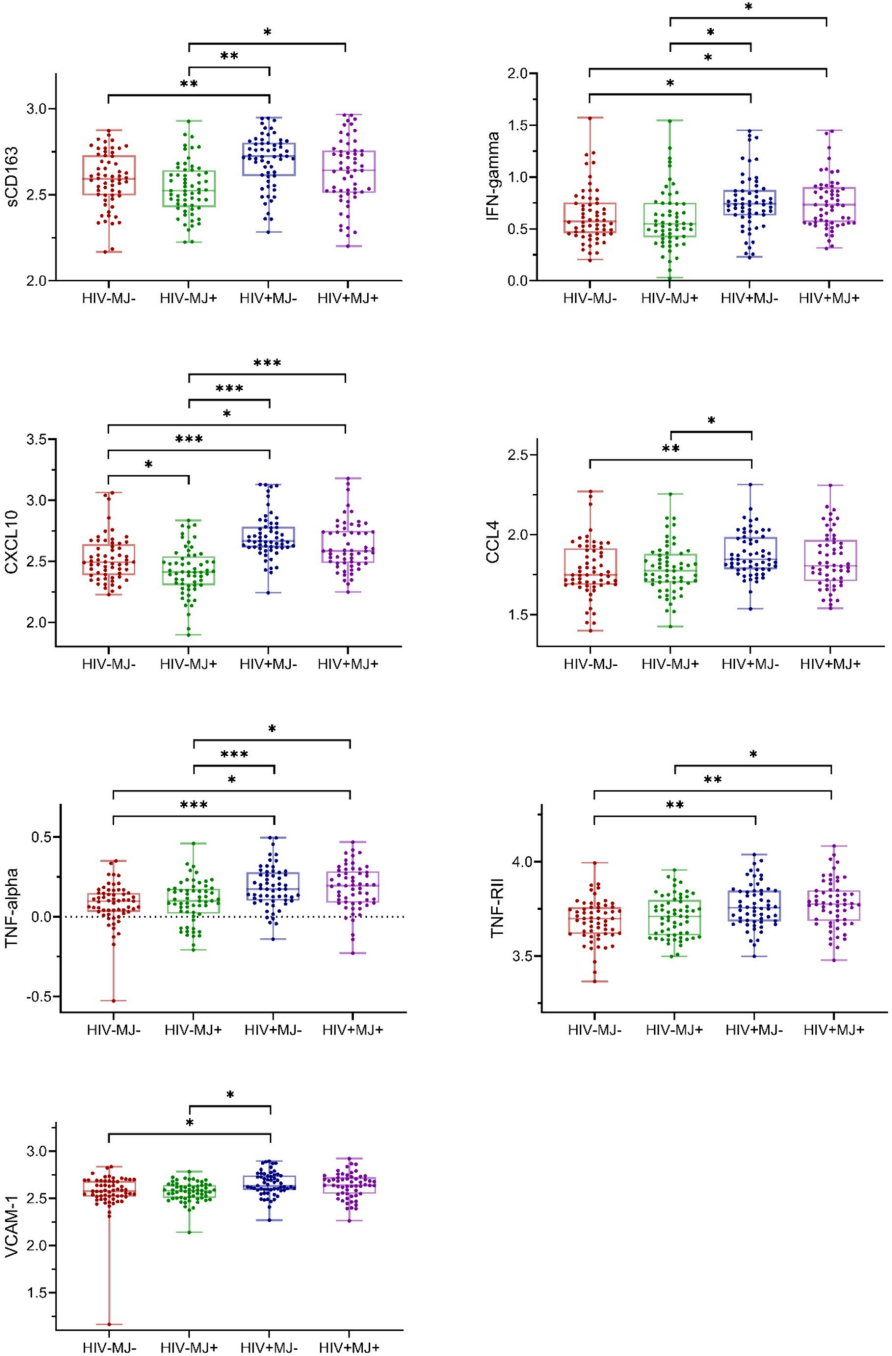
Adjusted pairwise comparisons plots for biomarkers of interest. Boxplots (median and interquartile range) with overlaid jittered individual observations show log-transformed biomarker values across the four HIV/marijuana-use groups. For each biomarker, a linear model was fit with Group as the primary predictor and adjusted for age, sex, alcohol use, nicotine use, kidney function, liver function, cardiometabolic function, and depression. Estimated marginal means were compared using pairwise contrasts, with *p*-values adjusted using the Benjamini-Hochberg (BH) procedure. Significant pairwise differences are annotated above the plots (*p < 0.05, ** *p* < 0.01, *** *p* < 0.001)

Figure 1 summarizes the pairwise comparisons of biomarker levels across the four HIV/MJ groups. Compared with HIV–MJ– participants, those in the HIV–MJ+ group had a significantly lower level of CXCL10; there were no other differences between these two groups. Participants with HIV who did not use marijuana (HIV + MJ–) showed significantly higher levels of monocyte/macrophage activation (sCD163), interferon signaling (IFN-γ), TNF-related inflammatory signaling (TNF-α, TNF-RII), chemokine signaling (CXCL10), and endothelial/trafficking activity (CCL4, VCAM-1) compared with HIV–MJ–, indicating broad elevations across activation, inflammatory, and trafficking-related markers

In the presence of both HIV and marijuana use (HIV + MJ+), markers of immune activation and inflammation (sCD163, IFN‑γ, TNF‑α, TNF‑RII, CXCL10) remained elevated relative to HIV–MJ–, similar in magnitude to the HIV + MJ– group. The only difference was that CCL4 and VCAM‑1, trafficking/endothelial markers, were no longer significantly elevated. However, direct comparisons between HIV + MJ+ and HIV + MJ– participants for CCL4 and VCAM-1 were not statistically significant.

### Correlations Between Biomarkers and Cannabinoid Metabolites

**Fig. 2:**
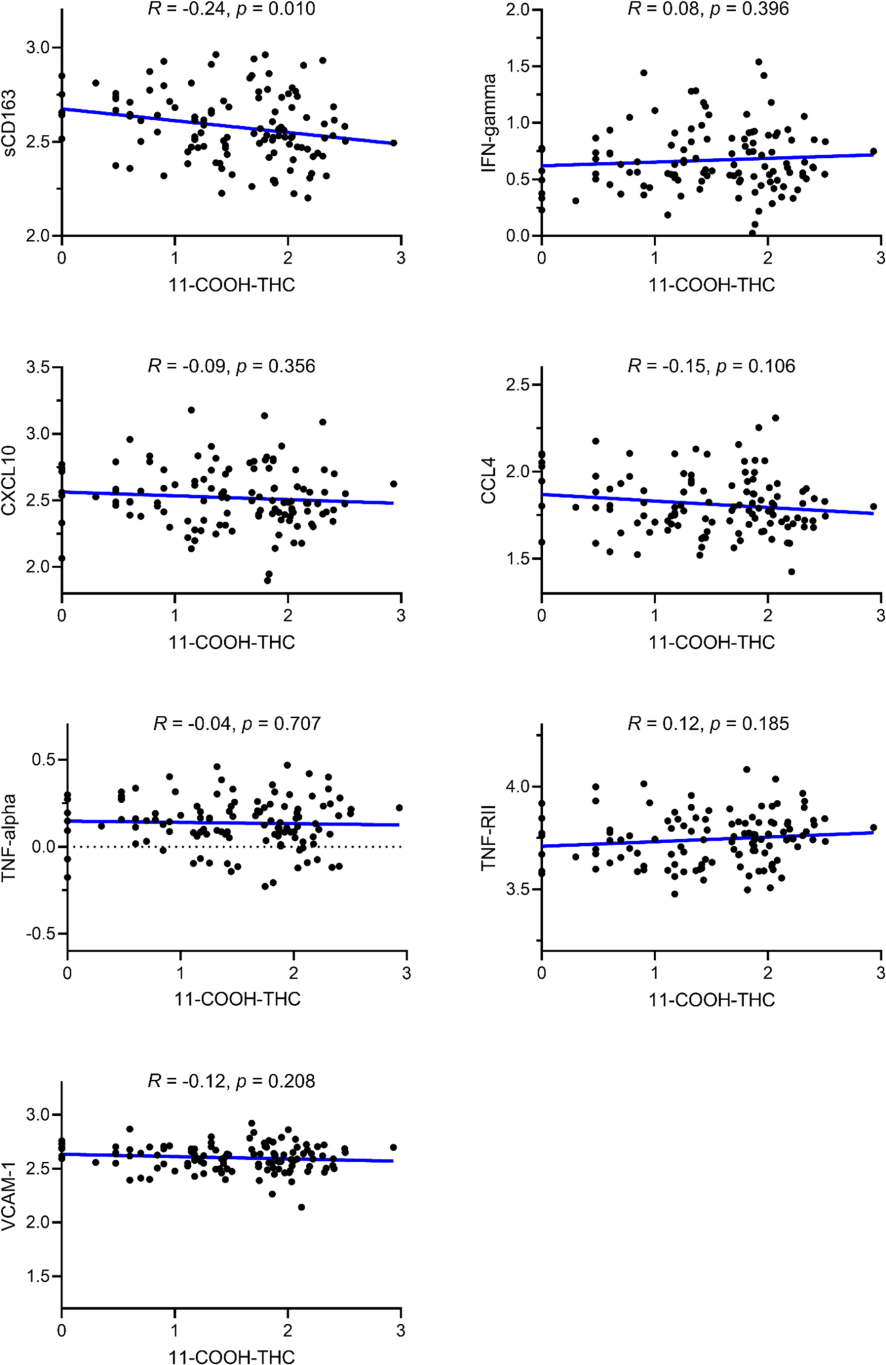
Adjusted pairwise comparisons plots for biomarkers of interest. Boxplots (median and interquartile range) with overlaid jittered individual observations show log-transformed biomarker values across the four HIV/marijuana-use groups. For each biomarker, a linear model was fit with Group as the primary predictor and adjusted for age, sex, alcohol use, nicotine use, kidney function, liver function, cardiometabolic function, and depression. Estimated marginal means were compared using pairwise contrasts, with *p*-values adjusted using the Benjamini-Hochberg (BH) procedure. Significant pairwise differences are annotated above the plots (*p < 0.05, ** *p* < 0.01, *** *p* < 0.001)

Among MJ+ participants, 11-COOH-THC concentrations showed a statistically significant but modest negative correlation with sCD163 (*r* = − 0.24, *p* = 0.010; Fig. 2). No associations were observed between 11-COOH-THC and the other six biomarkers identified in multivariable models. There was no moderation by HIV status. Correlations with 7-COOH-CBD were not evaluated due to low detection rates.

### Correlations Between Biomarkers and Cognitive Function

This analysis focused on the two cognitive domains in which there were group differences (learning and memory; Table 3). Memory scores were modestly negatively correlated with TNF‑α (*r* = − 0.16, *p* < 0.05) and TNF‑RII (*r* = − 0.16, *p* < 0.05), with no moderation by HIV status. No other biomarker-cognition correlations reached significance for either learning or memory.

**Table 3:** Correlations between biomarkers of interest, cognition T-scores (Learning and memory)

Biomarker/Metabolite	Cognition Domain	
	Learning T-Score	Memory T-Score
sCD163	0	-0.03
IFN-gamma	-0.05	-0.06
TNF-alpha	-0.09	-0.16*
TNF- RII	-0.09	-0.16*
CXCL10	-0.05	-0.08
CCL4	-0.04	-0.04
VCAM-1	-0.05	-0.07

Note: * = *p* < 0.05, ** = *p* < 0.01

## Discussion

This cohort study investigated whether marijuana use modifies HIV‑related immune activation and inflammatory signaling and evaluated how these biomarker profiles correspond to cognitive performance. Three major findings emerged: (1) among HIV‑negative participants, marijuana use was associated with a selective reduction in CXCL10, possibly due to alteration of IFN‑γ–CXCL10 chemotactic signaling rather than a broad shift in systemic inflammatory signaling; (2) HIV infection in the absence of marijuana use was associated with a coordinated, multi‑pathway signature of chronic immune activation and inflammation, including TNF signaling, Th1/interferon activation, monocyte/macrophage activation, and endothelial/adhesion programs that support leukocyte recruitment; and (3) among PWH, marijuana use did not attenuate HIV‑associated immune activation, with nearly all markers remaining elevated relative to HIV-negative participants (with the exception of CCL4 and VCAM-1).

Most participants used THC-dominant cannabis products, as indicated by the high prevalence of detectable 11-COOH-THC and minimal 7-COOH-CBD. This pattern is consistent with typical U.S. cannabis markets and suggests that the immunologic effects observed here are likely driven primarily by THC-related mechanisms rather than balanced THC/CBD exposure. Since cannabinoid effects on immune function could be dose- and context-dependent, our findings should not be interpreted as indicating global immunosuppression or protection. Instead, the pattern observed—selective changes in trafficking- and endothelial-related markers without broad reductions in inflammatory signaling—likely reflects the specific immunomodulatory profile of THC-dominant exposure in this cohort.

In individuals without HIV, the principal marijuana-associated change was a reduction in CXCL10, a chemokine induced downstream of IFNγ that binds CXCR3 and orchestrates chemotaxis and positioning of Th1-polarized T cells and other effector leukocytes toward inflamed tissues (Lei et al. 2019; Liu et al. 2011). CXCL10 is also a well-described component of inflammatory trafficking programs across infectious and immune-mediated contexts, and altered CXCL10 signaling can plausibly reduce the efficiency of immune cell recruitment to sites where pathogen control, debris clearance, and tissue repair are required (Liu et al. 2011). Consistent with this mechanism, human observational work in people with and without HIV has reported associations between cannabis exposure and lower CXCL10, supporting the plausibility that cannabinoids can modulate chemokine pathways relevant to HIV biology (Rogers et al. 2025; Watson et al. 2021). Functionally, this pattern is most consistent with trafficking dysregulation rather than global immunosuppression, raising the possibility that some infections or tissue injuries could experience delayed leukocyte arrival and slower inflammatory resolution when CXCL10-dependent recruitment is blunted (Liu et al. 2011).

An exploratory analysis revealed a negative association between sCD163 and 11-COOH-THC metabolite levels, which was not moderated by HIV status. While CD163 levels were not significantly altered by marijuana use grouping, higher THC exposure may be linked to reduced CD163 expression. A THC‑associated decrease in sCD163 may reflect cannabinoid‑driven shifts in macrophage activation state, microenvironmental signaling, or receptor shedding dynamics. While speculative, these mechanisms align with what is known about both THC’s immunomodulatory effects and the regulatory biology of CD163 (Fischer-Riepe et al. 2020; Skytthe et al. 2020).

In contrast, HIV infection in persons without MJ use produced a broad immunologic signature characteristic of persistent immune activation and inflammation. Elevated TNF-α and TNF-RII support sustained TNF-pathway activity, a central axis of chronic inflammation that is strongly linked to downstream immune activation and inflammatory tissue effects in treated and untreated HIV (Wilson et al., 2014). Increased IFN-γ with accompanying elevations of CXCL10 indicate persistent Th1/interferon-associated activation; importantly, CXCL10 is frequently elevated in HIV, correlates with disease activity in multiple studies, and has been associated with more rapid disease progression in early infection (Jiao et al. 2012; Lei et al. 2019; Valverde-Villegas et al. 2018). The increase in sCD163 further supports monocyte/macrophage activation as a major component of HIV-associated inflammation and comorbidity risk, including links to vascular disease and neurocognitive impairment (Burdo et al. 2011, 2013; Wilson et al., 2014). At the same time, CD163 biology is not purely pro-inflammatory: membrane CD163 is a hemoglobin–haptoglobin scavenger receptor enriched on alternatively activated macrophage states and contributes to anti-inflammatory/pro-resolution biology via heme uptake and the CD163–HO-1 pathway that generates anti-inflammatory metabolites (Kowal et al. 2011; Moestrup and Moller 2004; Thomsen et al. 2013). Thus, elevated sCD163 likely reflects both heightened macrophage activation and a compensatory, inflammation-buffering response that emerges under persistent inflammatory pressure; this duality is itself consistent with chronic inflammatory conditions where ongoing stimuli drive both inflammatory activation and counter-regulatory programs (Etzerodt and Moestrup 2013; Gaini et al. 2008). Finally, elevations in VCAM-1 and chemokines such as CCL4 align with enhanced endothelial activation and leukocyte recruitment capacity in HIV; soluble VCAM-1 is consistently elevated in HIV and relates to activation/inflammatory markers and disease dynamics (Graham et al. 2013; Melendez et al. 2008), supporting the concept that HIV shapes not only immune cell activation states but also vascular/adhesion environments that influence leukocyte trafficking.

When marijuana use was present in PWH, the HIV-associated activation phenotype persisted, but specific trafficking/endothelial markers (CCL4 and VCAM-1) did not show the same pattern of HIV-associated elevation observed in other markers. This pattern is coherent with experimental and clinical evidence that THC and CB1 receptor signaling can modulate endothelial activation and leukocyte transmigration/adhesion programs, including reductions in adhesion molecule expression and impaired leukocyte–endothelial interactions under inflammatory conditions. Experimental studies demonstrate that THC suppresses endothelial activation and leukocyte adhesion through CB1-dependent mechanisms, affecting pathways that regulate VCAM-1 expression and immune cell recruitment (Klein et al. 1998; Rajesh et al. 2007). Human studies also suggest cannabis exposure can associate with lower endothelial/chemokine biomarkers in certain contexts, including reports of cannabis-associated reductions in CXCL10 and conditional effects on VCAM-1 in cohorts stratified by HIV and other exposures (Rogers et al. 2025). In aggregate, these observations support a model in which marijuana does not primarily “reprogram” systemic inflammatory activation in HIV (which remains driven by chronic TNF/interferon/monocyte activation), but instead may influence pathways that regulated immune cell trafficking from the bloodstream into tissues—chemokine gradients, adhesion molecules, and endothelial-leukocyte interactions. A key biological implication is a potentially maladaptive “activated but poorly directed” immune state: HIV maintains systemically activated immune cells and inflammatory mediators, while alterations in trafficking/endothelial cues could hinder appropriate tissue homing, slowing resolution of inflammatory insults and potentially contributing to prolonged, compartmentalized dysfunction and chronification of comorbidity-relevant inflammation (Graham et al. 2013; Liu et al. 2011). This mechanistic mismatch is particularly relevant to HIV comorbidities because chronic immune activation and endothelial dysfunction are central contributors to cardiometabolic and neuroinflammatory risk; impaired recruitment and repair signaling could plausibly exacerbate persistence of inflammatory damage even without further amplification of systemic cytokine production (Burdo et al. 2011; Graham et al. 2013).

The group differences observed in learning and memory function may partially reflect the combined effects of HIV-driven immune activation and selective disruption of trafficking pathways that shape neuroimmune homeostasis. Persistent TNF signaling and monocyte/macrophage activation—hallmarks of HIV inflammation—are central to current models of HIV-associated neurocognitive disorders and can plausibly impair hippocampal-dependent learning and memory through neuroinflammatory effects on synaptic plasticity (Belarbi et al. 2012; Burdo et al. 2011; Prieto et al. 2019; Saylor et al. 2016). In our data, the modest negative correlations between TNF-α/TNF-RII and memory performance are consistent with this framework. Marijuana-associated changes, in contrast, mapped more strongly to trafficking/endothelial programs (reduced CXCL10 in HIV-MJ + and lack of CCL4/VCAM-1 elevation in HIV + MJ+), suggesting that cannabinoids may influence cognition partly by altering immune surveillance and resolution dynamics rather than by further amplifying systemic inflammation (Graham et al. 2013; Lei et al. 2019; Liu et al. 2011). Importantly, cognitive differences in marijuana users may also arise independently of peripheral inflammation via direct cannabinoid actions in the CNS: THC and related CB1 receptor agonists reliably impair verbal learning and memory, and mechanistic studies show CB1 signaling in hippocampal circuits (including astroglial CB1 pathways) is necessary for cannabinoid-related memory deficits (Han et al. 2012; Ranganathan et al. 2017; Wise et al. 2009; Zhornitsky et al. 2021). Thus, the learning/memory signal in HIV + MJ+ individuals may reflect both (i) an HIV-associated inflammatory milieu that increases cognitive vulnerability and (ii) CB1-mediated disruption of encoding and circuit plasticity, potentially compounded by altered immune/endothelial trafficking that could prolong low-grade neuroinflammatory stress.

### Strengths and Limitations

This study has several notable strengths. First, we employed a rigorous approach to stratify participants by HIV and marijuana use, allowing for a nuanced assessment of their associations with peripheral inflammation and cognition. Second, we used a comprehensive panel of peripheral biomarkers spanning multiple immune pathways, enhancing the biological interpretability of results. Third, we incorporated demographically adjusted T scores from validated neuropsychological instruments across multiple domains. Limitations include the cross‑sectional design, which limits causal inference about relationships between biomarkers, cognitive function, chronic HIV disease, and marijuana use. Generalizability may be constrained by the geographic region and sample characteristics, including a predominance of male and Black/African American participants. It may also be limited by the clinical profile of the HIV sample, as all PWH were virally suppressed on cART and had mean CD4 counts within the normal range, and thus may not reflect individuals with unsuppressed viremia or greater immunosuppression. Marijuana exposure was assessed using a combination of self-report, urine drug screening, and circulating cannabinoid levels; however, use was not standardized with respect to dose, potency, or route of administration, which may introduce measurement variability and limit inference regarding specific patterns of use. Finally, most participants used THC‑dominant products, suggesting that immunologic effects observed here are primarily attributable to THC exposure rather than balanced THC/CBD formulations.

#### Implications for Future Research

These findings underscore the need for future work to disentangle how cannabinoids interact with HIV-associated immune activation to shape trafficking biology, endothelial function, and neuroimmune outcomes. Longitudinal studies with well-characterized marijuana use—including product type, cannabinoid composition, frequency, route of administration, and biological verification via metabolites—will be essential for determining whether the selective attenuation of chemokine and adhesion pathways persists over time and influences trajectories of neurocognitive change. Mechanistic studies are also warranted to directly test whether cannabinoid exposure alters trafficking-relevant processes such as chemokine signaling, endothelial activation, and leukocyte adhesion or transmigration. Approaches could include ex vivo blood–brain barrier or microvascular endothelial models, in vitro monocyte/endothelial adhesion and migration assays, and functional interrogation of CXCL10- and VCAM-1–dependent pathways. Finally, multiomic profiling of monocytes, macrophages, and endothelial cells may help identify molecular programs through which cannabinoids differentially modulate inflammatory activation versus trafficking machinery, clarifying why marijuana appears to selectively influence recruitment and adhesion cues without broadly suppressing systemic cytokine signaling. Together, such efforts will advance understanding of how cannabinoids shape neuroimmune homeostasis in PWH and may help identify risk- or resilience-related pathways relevant to neuroHIV comorbidities.

## Conclusion

Our results suggest that marijuana use exerts selective, trafficking‑focused immunologic effects that differ substantially between people with and without HIV. In HIV‑negative individuals, marijuana selectively reduced CXCL10, suggesting targeted disruption of IFN‑γ–dependent chemotactic programs. HIV infection produced the expected multi‑pathway immune activation signature, and this inflammatory phenotype persisted in PWH who use marijuana; however, endothelial and leukocyte‑recruitment markers (CCL4 and VCAM‑1) did not show the same pattern of HIV-associated elevation. Notably, these markers did not differ between PWH with and without marijuana use, suggesting that this pattern may reflect divergence from the broader HIV-associated activation profile rather than a clear marijuana-associated attention within PWH. This pattern supports a model in which cannabinoids modulate immune cell mobilization and endothelial–immune interfaces rather than systemic inflammatory signaling. Given the importance of trafficking and endothelial biology in neuroimmune homeostasis, these selective alterations may contribute to cognitive vulnerability through combined effects on inflammatory signaling, leukocyte recruitment, and cannabinoid‑mediated modulation of hippocampal circuits.

## Supplementary Information

Below is the link to the electronic supplementary material.

Supplementary Material 1

## Data Availability

The data that supports the findings of this study are available from the corresponding author upon reasonable request.
