# Supplementary material for: Neuroimmune Correlates of HIV and Marijuana Use: Peripheral Biomarkers and Cognitive Function
Source: J Neuroimmune Pharmacol. 2026 Jun 23;21(1):31. doi: 10.1007/s11481-026-10299-6 (PMC13287204; doi:10.1007/s11481-026-10299-6)
Supplement: Supplementary file 1 — Supplementary Material 1 [file 11481_2026_10299_MOESM1_ESM.docx]

**Supplemental Table 1.** Neuropsychological Assessments by Domain

| **Domain** | **Tests** |
| --- | --- |
| Executive Function | D-KEFS Tower Test;^1^ Wisconsin Card Sorting Test,^2^ Stroop Interference;^3^ Trail Making B.^4,5^ |
| Working Memory | Paced Auditory Serial Addition;^5^ WAIS-IV Digit Span and Letter-Number Sequencing;^6^ WMS-IV Spatial Addition.^7^ |
| Motor Ability | Grooved Pegboard;^4,5^ Finger Tapping (dominant and non-dominant hands).^4,5^ |
| Learning | Hopkins Verbal Learning Trials 1-3;^9^ Brief Visual Memory Trials 1-3;^10^ WMS-IV Logical Memory I.^8^ |
| Memory | Hopkins Verbal Learning Trial 4;^9^ Brief Visual Memory Trial 4;^10^ WMS-IV Logical Memory II.^8^ |
| Processing Speed | Trail Making – Part A;^4^ WAIS-IV Coding; 104 Stroop Color Naming.^3^ |
| Fluency | Controlled Oral Word Association (FAS and animals);^5,11^ D-KEFS Design Fluency^1^ |

1. Delis DC, Kaplan E, Kramer JH. Delis–Kaplan Executive Function System (D-KEFS): Examiner's manual. San Antonio, TX: The Psychological Corporation; 2001.
2. Kongs SK, Thompson LL, Iverson GL, Heaton RK. Wisconsin Card Sorting Test-64 Computerized Version. Odessa, FL: Psychological Assessment Resources; 2000.
3. Golden CJ. Stroop Color and Word Test. Chicago, IL: Stoelting; 1978.
4. Reitan RM, Wolfson D. The Halstead-Reitan neuropsychological test battery: Theory and clinical interpretation 2nd ed. Tucson, AZ: Neuropsycholgy Press 1993.
5. Heaton RK, Grant I, Matthews CG. Comprehensive Norms for an Expanded Halstead-Reitan Battery: Demographic Corrections, Research Findings, and Clinical Applications. Odessa, FL: Psychological Assessment Resources; 1991.
6. Diehr MC, Cherner M, Wolfson TJ, Miller SW, Grant I, Heaton RK. The 50 and 100-item short forms of the Paced Auditory Serial Addition Task (PASAT): Demographically corrected norms and comparisons with the full PASAT in normal and clinical samples. J Clin Exp Neuropsychol. 2003;25:571-585.
7. Wechsler D. Wechsler Adult Intelligence Scale -- Fourth Edition. San Antonio, TX: Pearson Assessment; 2008.
8. Wechsler D. Wechsler Memory Scale -- Fourth Edition. San Antonio, TX: Pearson Assessment; 2009.
9. Brandt J, Benedict RHB. Hopkins Verbal Learning Test -- Revised professional manual. Lutz, FL: Psychological Assessment Resources, Inc; 2001.
10. Benedict RHB, Schretlen D, Groninger L, Dobraski M. Revision of the Brief Visuospatial Memory Test: studies of normal performance, reliability, and validity. Psychol Assess. 1996;8:145-153.
11. Benton A, Hamsher K, Sivan A. Multilingual Aphasia Examination (3rd ed.). Iowa City, IA: AJA Associates; 1983.

**Supplemental Table 2.** Full Model Results for Biomarkers Selected for Further Comparison

| **Model Variables** | **Beta estimate** | ***p*-value** | **FDR-corrected *p*-value** |
| --- | --- | --- | --- |
| **sCD163** |  |  |  |
| ***Intercept*** | 2.440 | **<0.001** | **<0.001** |
| ***HIV/MJ Group*** |  |  |  |
| HIV-MJ- | REF | REF | REF |
| HIV-MJ+ | -0.022 | 0.525 | 0.802 |
| HIV+MJ- | 0.108 | **0.001** | **0.005** |
| HIV+MJ+ | 0.059 | 0.094 | 0.259 |
| ***Age*** | 0.004 | **0.009** | **0.042** |
| ***Sex*** |  |  |  |
| Female | REF | REF | REF |
| Male | -0.006 | 0.804 | 0.947 |
| ***Alcohol*** | 0.000 | 0.949 | 0.994 |
| ***Nicotine in Past 30 Days*** |  |  |  |
| No | REF | REF | REF |
| Yes | -0.055 | 0.058 | 0.188 |
| ***Kidney Function*** |  |  |  |
| Normal | REF | REF | REF |
| Abnormal | -0.009 | 0.698 | 0.873 |
| ***Liver Function*** |  |  |  |
| Low Risk | REF | REF | REF |
| Elevated Risk | 0.002 | 0.970 | 0.994 |
| ***High Cholesterol*** |  |  |  |
| No | REF | REF | REF |
| Yes | -0.036 | 0.239 | 0.502 |
| ***Depression SCL Score*** | -0.012 | 0.600 | 0.855 |
| **IFN-gamma** |  |  |  |
| ***Intercept*** | 0.574 | **<0.000** | **<0.000** |
| ***HIV/MJ Group*** |  |  |  |
| HIV-MJ- | REF | REF | REF |
| HIV-MJ+ | -0.016 | 0.783 | 0.940 |
| HIV+MJ- | 0.142 | **0.007** | **0.039** |
| HIV+MJ+ | 0.130 | **0.029** | 0.109 |
| ***Age*** | 0.002 | 0.461 | 0.759 |
| ***Sex*** |  |  |  |
| Female | REF | REF | REF |
| Male | -0.016 | 0.717 | 0.873 |
| ***Alcohol*** | -0.001 | 0.621 | 0.869 |
| ***Nicotine in Past 30 Days*** |  |  |  |
| No | REF | REF | REF |
| Yes | -0.023 | 0.637 | 0.873 |
| ***Kidney Function*** |  |  |  |
| Normal | REF | REF | REF |
| Abnormal | -0.040 | 0.302 | 0.619 |
| ***Liver Function*** |  |  |  |
| Low Risk | REF | REF | REF |
| Elevated Risk | -0.002 | 0.976 | 0.994 |
| ***High Cholesterol*** |  |  |  |
| No | REF | REF | REF |
| Yes | -0.003 | 0.948 | 0.994 |
| ***Depression SCL Score*** | 0.021 | 0.590 | 0.855 |
| **TNF-alpha** |  |  |  |
| ***Intercept*** | -0.023 | 0.692 | 0.873 |
| ***HIV/MJ Group*** |  |  |  |
| HIV-MJ- | REF | REF | REF |
| HIV-MJ+ | -0.002 | 0.951 | 0.994 |
| HIV+MJ- | 0.0988 | **<0.000** | **0.001** |
| HIV+MJ+ | 0.0712 | **0.014** | 0.057 |
| ***Age*** | 0.002 | 0.228 | 0.502 |
| ***Sex*** |  |  |  |
| Female | REF | REF | REF |
| Male | 0.016 | 0.457 | 0.759 |
| ***Alcohol*** | -0.000 | 0.848 | 0.976 |
| ***Nicotine in Past 30 Days*** |  |  |  |
| No | REF | REF | REF |
| Yes | 0.043 | 0.063 | 0.197 |
| ***Kidney Function*** |  |  |  |
| Normal | REF | REF | REF |
| Abnormal | 0.026 | 0.165 | 0.403 |
| ***Liver Function*** |  |  |  |
| Low Risk | REF | REF | REF |
| Elevated Risk | 0.027 | 0.502 | 0.802 |
| ***High Cholesterol*** |  |  |  |
| No | REF | REF | REF |
| Yes | 0.030 | 0.233 | 0.502 |
| ***Depression SCL Score*** | 0.015 | 0.437 | 0.759 |
| **TNF- RII** |  |  |  |
| ***Intercept*** | 3.578 | **<0.000** | **<0.000** |
| ***HIV/MJ Group*** |  |  |  |
| HIV-MJ- | REF | REF | REF |
| HIV-MJ+ | 0.028 | 0.233 | 0.502 |
| HIV+MJ- | 0.077 | **0.001** | **0.005** |
| HIV+MJ+ | 0.081 | **0.001** | **0.009** |
| ***Age*** | 0.002 | 0.056 | 0.187 |
| ***Sex*** |  |  |  |
| Female | REF | REF | REF |
| Male | 0.015 | 0.423 | 0.759 |
| ***Alcohol*** | -0.003 | **0.004** | **0.023** |
| ***Nicotine in Past 30 Days*** |  |  |  |
| No | REF | REF | REF |
| Yes | 0.018 | 0.369 | 0.689 |
| ***Kidney Function*** |  |  |  |
| Normal | REF | REF | REF |
| Abnormal | 0.028 | 0.085 | 0.247 |
| ***Liver Function*** |  |  |  |
| Low Risk | REF | REF | REF |
| Elevated Risk | 0.003 | 0.942 | 0.994 |
| ***High Cholesterol*** |  |  |  |
| No | REF | REF | REF |
| Yes | 0.009 | 0.674 | 0.873 |
| ***Depression SCL Score*** | 0.016 | 0.335 | 0.655 |
| **CXCL10** |  |  |  |
| ***Intercept*** | 2.428 | **<0.000** | **<0.000** |
| ***HIV/MJ Group*** |  |  |  |
| HIV-MJ- | REF | REF | REF |
| HIV-MJ+ | -0.102 | **0.009** | **0.042** |
| HIV+MJ- | 0.177 | **<0.000** | **<0.000** |
| HIV+MJ+ | 0.106 | **0.009** | **0.042** |
| ***Age*** | 0.003 | 0.168 | 0.403 |
| ***Sex*** |  |  |  |
| Female | REF | REF | REF |
| Male | -0.027 | 0.366 | 0.689 |
| ***Alcohol*** | -0.001 | 0.451 | 0.759 |
| ***Nicotine in Past 30 Days*** |  |  |  |
| No | REF | REF | REF |
| Yes | -0.026 | 0.429 | 0.759 |
| ***Kidney Function*** |  |  |  |
| Normal | REF | REF | REF |
| Abnormal | 0.026 | 0.326 | 0.653 |
| ***Liver Function*** |  |  |  |
| Low Risk | REF | REF | REF |
| Elevated Risk | 0.074 | 0.179 | 0.419 |
| ***High Cholesterol*** |  |  |  |
| No | REF | REF | REF |
| Yes | -0.013 | 0.717 | 0.873 |
| ***Depression SCL Score*** | 0.005 | 0.863 | 0.979 |
| **CCL4** |  |  |  |
| ***Intercept*** | 1.620 | **<0.000** | **<0.000** |
| ***HIV/MJ Group*** |  |  |  |
| HIV-MJ- | REF | REF | REF |
| HIV-MJ+ | 0.014 | 0.655 | 0.873 |
| HIV+MJ- | 0.096 | **0.001** | **0.010** |
| HIV+MJ+ | 0.0676 | **0.046** | 0.162 |
| ***Age*** | 0.003 | **0.027** | 0.109 |
| ***Sex*** |  |  |  |
| Female | REF | REF | REF |
| Male | 0.0506 | **0.042** | 0.155 |
| ***Alcohol*** | -0.003 | 0.096 | 0.259 |
| ***Nicotine in Past 30 Days*** |  |  |  |
| No | REF | REF | REF |
| Yes | 0.014 | 0.600 | 0.855 |
| ***Kidney Function*** |  |  |  |
| Normal | REF | REF | REF |
| Abnormal | 0.000 | 0.994 | 0.994 |
| ***Liver Function*** |  |  |  |
| Low Risk | REF | REF | REF |
| Elevated Risk | -0.070 | 0.127 | 0.324 |
| ***High Cholesterol*** |  |  |  |
| No | REF | REF | REF |
| Yes | 0.002 | 0.952 | 0.994 |
| ***Depression SCL Score*** | -0.014 | 0.521 | 0.802 |
| **VCAM-1** |  |  |  |
| ***Intercept*** | 2.422 | **<0.000** | **<0.000** |
| ***HIV/MJ Group*** |  |  |  |
| HIV-MJ- | REF | REF | REF |
| HIV-MJ+ | 0.003 | 0.924 | 0.994 |
| HIV+MJ- | 0.088 | **0.003** | **0.017** |
| HIV+MJ+ | 0.056 | 0.084 | 0.247 |
| ***Age*** | 0.002 | 0.117 | 0.307 |
| ***Sex*** |  |  |  |
| Female | REF | REF | REF |
| Male | 0.064 | **0.007** | **0.039** |
| ***Alcohol*** | -0.001 | 0.559 | 0.838 |
| ***Nicotine in Past 30 Days*** |  |  |  |
| No | REF | REF | REF |
| Yes | 0.011 | 0.690 | 0.873 |
| ***Kidney Function*** |  |  |  |
| Normal | REF | REF | REF |
| Abnormal | -0.000 | 0.988 | 0.994 |
| ***Liver Function*** |  |  |  |
| Low Risk | REF | REF | REF |
| Elevated Risk | 0.031 | 0.509 | 0.802 |
| ***High Cholesterol*** |  |  |  |
| No | REF | REF | REF |
| Yes | -0.007 | 0.812 | 0.947 |
| ***Depression SCL Score*** | 0.009 | 0.664 | 0.873 |
